# Wireless patient monitoring and Efficacy Safety Score in postoperative treatment at the ward: evaluation of time consumption and usability

**DOI:** 10.1007/s10877-023-01053-x

**Published:** 2023-07-17

**Authors:** Erlend Johan Skraastad, Petter Christian Borchgrevink, Lillian Asbøll Opøyen, Johan Ræder

**Affiliations:** 1 Clinic of Anaesthesia and Intensive Care, St. Olavs hospital, Trondheim University Hospital, 3250 Torgarden, 7006 Trondheim, Norway; 2 Department of Circulation and Medical Imaging, Faculty of Medicine and Health Sciences, Norwegian University of Science and Technology, Trondheim, Norway; 3 Unit on Complex Symptom Disorders, St. Olavs hospital, Trondheim University Hospital, Trondheim, Norway; 4 Department of Thoracic and Occupational Medicine and Orkdal Dept. of Internal Medicine, St. Olavs Hospital, Trondheim University Hospital, Trondheim, Norway; 5 Department of Anaesthesia and Intensive Care Medicine, Oslo University Hospital, Oslo, Norway; 6 Institute for Clinical Medicine, Medical Faculty, University of Oslo, Oslo, Norway

**Keywords:** Patient safety, Postoperative care, Nursing, Point-of-care systems, Decision making

## Abstract

To evaluate objective time consumption and how nurses perceived introducing wireless patient monitoring (WPM) and a validated score on patient quality and safety, the Efficacy Safety Score (ESS), at a mixed surgery ward. After fulfilling a randomised controlled trial combining the ESS and WPM, we addressed time consumption and conducted a questionnaire survey among nurses who participated in the study. The questionnaire appraised the nurses’ evaluation of introducing these tools for postoperative management. Of 28 invited nurses, 24 responded to the questionnaire, and 92% reported the ESS and WPM-systems to increase patient safety and quality of care. 67% felt the intervention took extra time, but objective workload measurements revealed reduced time to 1/3 using ESS and WPM compared to standard manual assessment. Improved confidence when using the systems was reported by 83% and improved working situation by 75%. In a test situation to measure time consumption, the ESS and pre-attached WPM-systems require less time than the conventional standard of care, and may allow for more frequent clinical monitoring at the post-surgical ward. The combination of the ESS and the WPM systems was perceived as positive by participating nurses and further clinical development and research is warranted.

**Supplementary Information:**

The online version contains supplementary material available at 10.1007/s10877-023-01053-x.

## Background

Postoperative care involves patient safety, patient-perceived quality, cost-efficacy, and personnel satisfaction. The nurses’ responsibility is to do first-line safety and quality assessments, deliver medication, evaluate the effect and potential side effect(s), and document patient measures and outcomes as a part of postoperative care.

Developing effective and reliable systems for postoperative nursing care is essential to reach optimal care after surgery [1, 2]. However, as the staffing is less numerous at the ward than in the postoperative care unit, delivering high-quality care in this environment is challenging. Therefore, simple but still specific and sensitive tools are needed [3, 4].

Early warning scores for safety issues, i.e., identification of medical deterioration, with a different design for different patient categories, are widely used [5]. Further, simple objective outcomes after surgery, such as drinking, eating, and mobilising, are used to measure postoperative quality [6]. At our surgical wards, the National Early Warning Score (NEWS) is in routine use, based on pen and paper registration by the nurses. The NEWS system has been a part of mandatory ward care at our hospital since 2016.

Effective pain treatment and anti-emetic measures are crucial to achieving functional outcomes and reducing anxiety, discomfort, and post-surgery stress responses [7]. Unfortunately, despite clinical guidelines and multimodal approaches for adequate pain management regimes, pain is undertreated in daily practice [8, 9].

Further, there has been a need for a medical device to simplify postoperative monitoring since nurses spend about 30 min to an hour on clinical documentation for every hour spent on patient care [10]. Introducing electronic medical record point of care documentation in patients’ rooms is shown to be less time consuming and beneficial to patient care [11]. In addition, the quality of patient care may improve with the standardisation, implicit with automatic electronic nursing documentation [12]. Studies have shown that new technology for remote and wireless patient monitoring (WPM) can increase patient safety and quality without interfering with early mobilisation [13, 14].

For these reasons, we have developed and documented the successful use of the Efficacy Safety Score (ESS) as an alternative, complete tool for nurse surveillance of safety and quality on postoperative patients for daily, routine use at the ward. The ESS consists of five clinical features regarding the patient’s postoperative status: mental condition, postoperative nausea and vomiting (PONV), pain at rest, pain at movement and general condition. Depending on the patient’s status or complaints, each clinical feature is scored from 0 to 15 and summarised in a total score. ESS ≥ 10 is suggested as an appropriate cut-off value for problems in need of someone staying with the patient as well as immediate consultation with a physician [15].

The introduction of the ESS as a decision tool combined with a wireless patient monitoring (WPM) system has previously been tested out in a randomised controlled trial (RCT) by our group in a clinical postoperative ward setting [16]. The results showed less pain, increased satisfaction and more rapid mobilisation for patients using ESS and WPM than standard care.

The objectives of the present study were to evaluate how nurses on the ward perceived this combination of the ESS and the WPM in terms of improving patient care, reducing time consumption, and improving interaction with patients and doctors.

## The study

### Design

We chose a descriptive design using a questionnaire to get information about the nurses’ experience introducing new technology as part of a randomised trial with a control group. The study was done in a mixed surgery ward for acute and elective abdominal, urological and orthopaedic surgery with 26 beds at a Norwegian university hospital, evaluating the ESS combined with a WPM for postoperative management. The patients’ safety and quality results have previously been reported [16]. This nurse survey started five weeks after the RCT period had ended. The nurses were unaware of the results of the RCT regarding patient data when they completed the survey. All the nurses working at the ward have prior experience using wireless monitoring devices in the form of cardiac telemetry.

The senior charge nurse provided a list of the 30 nurses working at the ward during the RCT period. This list was the basis for the online survey distribution made by the hospital’s license holder for the Questback survey solution (Questback GmbH. Version EFS Winter 2018, Cologne, Germany).

### The randomised controlled trial settings

The basis for this survey was the RCT period from March 15th to October 18th 2018, with 195 patients randomised into two groups; ESS and WPM intervention (INT-Group) versus standard care (SC-Group), described in detail in our previous report [16]. The nurses got a 45 min lecture about ESS and postoperative pain treatment before the start of the RCT. They also got a 15 min practical introduction and education using the WPM system.

For the INT-Group patients, the nurses assessed ESS in parallel with electronic automatic retrieved vital signs from wireless and wearable sensors [13] with the platform Patient Status Engine (PSE). The PSE is a class IIa CE-marked medical WPM device (Isansys Lifecare Ltd., Oxfordshire, U.K.) for hospital use, monitoring heart rate, ECG, ventilation rate, axillar skin temperature, blood pressure (initiated manually) and finger pulse oximetry. The bedside tablet PSE also gives an updated NEWS every minute, and the nurses stored the registered ESS on this PSE platform.

For the SC-Group patients, NEWS was documented on paper formularies at least every 12 h or with increased frequency in the presence of increased symptom severity.

All actual adverse events in the patients from both groups were registered during the RCT study period.

### Financial support

The ESS is developed with support from The Norwegian Medical Association’s foundation for quality improvement and patient safety. In 2018, the principal investigator received a two-year scholarship from The Joint Research Committee between St. Olavs hospital and the Faculty of Medicine and Health Sciences, NTNU.

### Methods

The authors developed the nurse surveillance questionnaire (see addendum) based on three former validated questionnaires for electronic information platforms. These were: a translated version of the Intranet Satisfaction Questionnaire [17], the Norwegian version of a questionnaire for evaluating electronic medical records systems [18] and the System Usability Scale [19], adapted to fit with our specific clinical setting and questions of interest.

All relevant items from these three questionnaires were modified through a stepwise process using internal and external expertise of three anaesthesiologists and two senior nurses. This process resulted in an extension of some items with additional questions about the quality of patient care, patient safety, perceived patient satisfaction with the intervention system, the collaboration between health care professionals, and estimated time consumption. In addition, this process excluded some items on specific technical support. The expert group also evaluated the readability, language, and clarity of wording in the questionnaire.

The final questionnaire consisted of nine background questions and 16 usability-focused questions, of which 14 questions had a structured response format with responses provided in a dichotomous (yes/no) or five-point Likert scale: Strongly agree (SA), Agree (A), Neutral (0), Disagree (DA) and Strongly disagree (SD). Two questions were open-ended (see Attachment). We analysed all the answers from the usability part of the questionnaire for this report.

Also, after finishing the RCT, we immediately set up a standardised test to measure time consumption and workload for the intervention (ESS and WPM) versus standard vital measurements (NEWS). Ten nurses who had participated in the RCT, all working on a randomly selected day, performed the different measurements and scores on two volunteer patients. They used the same equipment in the same environment and setting as in the RCT, where the patients arrived at the ward with the WPM-monitoring already attached and connected. We measured the time from starting the assessment or unlocking the monitor until completing documentation. We conducted the survey according to the Declaration of Helsinki standards, and the report follows Standards for Quality Improvement Reporting Excellence (SQUIRE 2.0) [20].

### Analysis

To summarise the questionnaire items, we used descriptive statistics. The questions were: how satisfied or dissatisfied are you with: (1) the wireless equipment and (2) the screen layout. Years of age and experience were recoded into five and six categories, respectively.

We registered all the collected data in Microsoft® Excel® for PC, version 16, and used SPSS version 26.0 (SPSS, Chicago, IL, USA) for data analysis.

### Ethics

The local hospital administration and the Regional Committee for Medical and Health Research Ethics (reference number 2017/1903/REK South East A) approved the survey, as part of a protocol registered at clinicaltrials.gov (NCT03438578) for validation of novel tools for postoperative management. Participation in the survey was voluntary, and all responses were anonymous. Informed consent was obtained from all participants and volunteers in the survey.

## Results

The participants completed the survey between Nov 30th, 2018 and Feb 6th, 2019. Thirty nurses were potential respondents to this study, but two had not used the intervention. Four did not respond to the survey, resulting in a response rate of 86%. Thus, we included 24 nurses reporting to have treated patients both in the INT-Group and SC-Group during the RCT period.

The demographics of age, gender and work experience are shown in Table 1.

**Table 1:** Demographics

Sex	Female		Male			
n = 24 (percent)	21 (87.5)		3 (12.5)			

Age, years	20–29	30–39	40–49	50–59	> 60	Not answered
n = 24 (percent)	6 (25.0)	4 (16.7)	5 (20.8)	6 (25.0)	1 (4.2)	2 (8.3)

Work experience as nurse, years	0–4	5–9	10–14	15–19	20–24	> 25
n = 24 (percent)	5 (20.8)	2 (8.3)	3 (12.5)	5 (20.8)	3 (12.5)	6 (25.0)

During the period of the RCT, the mean number of patients treated by each nurse in the SC-Group and INT-Group were 3.5 and 3.4, respectively. The mean numbers of documented sessions of standard care or intervention for each nurse were 11.3 and 20.8 times, respectively. In the usability part of the survey with 24 nurses, almost all agreed (46% somewhat and 46% strongly) upon the statement that the ESS and WPM intervention increased patient safety and provided better postoperative quality for patients than standard care (Table 2). 58% agreed that the intervention improved medical collaboration with physicians, while 8% disagreed. 83% of the nurses reported that the ESS and WPM intervention improved their confidence, and 92% said the patients perceived the intervention as positive. 67% felt the ESS and WPM took extra time compared to standard practice. However, 75% felt the intervention improved their overall working situation. Almost all (i.e., 96%) wanted to continue with the systems after the end of the study.

**Table 2:** Answers from the usability part of questionnaire survey

	Strongly disagree	Somewhat disagree	Neutral		Somewhat agree		Strongly agree
Monitoring with ESS and WPM provide increased safety for the patients (n = 24)	0 (0.0%)	0 (0.0%)	2 (8.3%)		11 (45.8%)		11 (45.8%)
Monitoring with ESS and WPM provide better post-operative quality for patients compared to standard practice (n = 24)	0 (0.0%)	0 (0.0%)	2 (8.3%)		11 (45.8%)		11 (45.8%)
Monitoring with ESS and WPM improves collaboration with physicians on measures and regulations for medicines (n = 24)	1 (4.2%)	1 (4.2%)	8 (33.3%)		11 (45.8%)		3 (12.5%)
Monitoring with ESS and WPM is perceived as a good measure by the patients (n = 24)	0 (0.0%)	0 (0.0%)	2 (8.3%)		9 (37.5%)		13 (54.2%)
Monitoring with ESS and WPM provides improved confidence for me as a nurse (n = 24)	0 (0.0%)	1 (4.2%)	3 (12.5%)		11 (45.8%)		9 (37.5%)
Monitoring with ESS and WPM takes extra time compared to normal practice (n = 24)	1 (4.2%)	5 (20.8%)	2 (8.3%)		10 (41.7%)		6 (25.0%)
Monitoring with ESS and WPM improves my overall working situation. (n = 24)	1 (4.2%)	1 (4.2%)	4 (16.7%)		14 (58.3%)		4 (16.7%)
	Very dissatisfied	Dissatisfied	Neutral		Satisfied		Very satisfied
How satisfied or dissatisfied are you with the WPM equipment? (n = 24)	1 (4.2%)	4 (16.7%)	9 (37.5%)		10 (41.7%)		0 (0.0%)
How satisfied or dissatisfied are you with the WPM screen layout? (n = 24)	0 (0.0%)	0 (0.0%)	7 (29.2%)		16 (66.7%)		1 (4.2%)
How satisfied or dissatisfied are you with the WPM training? (n = 24)	0 (0.0%)	3 (12.5%)	11 (45.8%)		7 (29.2%)		3 (12.5%)
How satisfied or dissatisfied are you with ESS training? (n = 24)	1 (4.2%)	3 (12.5%)	13 (54.2%)		5 (20.8%)		2 (8.3%)
				Yes		No	
Have you experienced that ESS and WPM have not detected medical deteriorated patients? (n = 24)				3 (12.5%)		21 (87.5%)	
Have you had medical deteriorated patients you think should have been monitored with ESS and WPM? (n = 24)				15 (62.5%)		9 (37.5%)	
Do you want to continue with ESS and WPM at the ward? (n = 24)				23 (95.8%)		1 (4.2%)	

In the simulated situation on the volunteers, the mean time for performing the ESS assessment was 86 (95% CI 75–97) seconds (n = 8), significantly faster than 274 (95% CI 229–319) seconds (n = 10) for manually performing the NEWS (p < 0.001).

The mean number of completed patient registrations from our previous report on patient data during 24 h was 8.2 vs. 3.4 times for the INT-Group (i.e., ESS and WPM) and the SC-Group (i.e., manually NEWS), respectively [16].

When extrapolating the time consumption data from the present simulation to these previously reported data, the result is a mean of 704 (95% CI 612–797) seconds for the total time used per patient in the INT-Group versus 931 (95% CI 779–1984) seconds for the SC-Group, a difference of 227 (95% CI 59–395) seconds, p = 0.011.

Regarding satisfaction with the electronic WPM equipment, 21% reported not being satisfied, 38% were neutral, and 42% were satisfied. There were twelve comments on the equipment, five about the irregularity of synchronisation of blood pressure monitor and the tablet, three about problems with finger saturation probes causing inconsistent measurements, and six general comments on the equipment being cumbersome and not user-friendly. Three participants commented in the questionnaire, having experienced that the WPM did not detect medical deteriorated patients. In these three cases, the nurses reported technical problems, all recognised by the nurses and handled using available equipment. One case was an impaired signal from the wireless skin temperature sensor and was solved using manual control of ear temperature. The second was a lack of wireless connection between the tablet and blood pressure monitor, which was solved using a manual blood pressure measurement device. The third case was a loss of battery power in the finger saturation probe due to failed replacement of batteries. This latter case was detected by the system’s warning and solved using a conventional finger pulse oximeter and manual registration.

One nurse felt the whole setup and equipment needed more development before being used as a routine. Another four nurses felt the training could be improved but still wanted continued use at their ward, as did the remaining 19 nurses.

## Discussion

The main findings in this study were that the nurses perceived introducing the ESS and WPM to improve the safety and quality for the patients and the working conditions at the ward. They also used only 1/3 of the time using the ESS and WPM-systems compared to manual assessment and documentation in the test setting. This setting simulated the clinical setting where the WPM-sensors are attached to the patient and paired with the PSE tablet prior to ward arrival. They also reported improved confidence and improved collaboration with physicians.

The finding of almost all the nurses reported increased patient safety by using these combined systems agrees with earlier reported perceptions from point-of-care electronic bedside charting and continuous vital sign measurements [11, 21]. These studies show that point-of-care documentation reduces the probability of errors. Further, automated advisory monitors reduce the time required by nurses to measure vital signs, which in turn leads to a quicker response when needed. The nurses’ opinion of increased patient safety is further consistent with the findings of the underlying RCT [16]. The ESS and WPM identified postoperative patients at risk of deterioration: two patients with hypotension and one with atrial fibrillation, compared to none reported in the standard care group. In the standard care group, two patients experienced major complications: one was accidentally found with extensive bradycardia and a seizure, and another had a stroke after mobilisation. The routine monitoring did not register these complications. Further, twice as many patients in the intervention group got supplementary oxygen at the ward, indicating improved identification of clinical deterioration. The ESS includes using a call-out algorithm if ESS ≥ 10, that facilitates early intervention in medical deterioration [15].

Another finding in this study consistent with the results in the underlying RCT [16] is that almost all nurses agreed that monitoring with ESS and WPM provides better postoperative quality for patients than standard practice. In the RCT, the rate of mobilisation was 54% higher for intervention patients than standard patients at any given time-point studied.

Improved pain relief can be an important part of the nurses’ perception of improved postoperative quality of care. The results from the RCT with the INT-Group patients reporting lower mean average intensity compared to the SC-Group patients is consistent with this. Further, the mean number of documented pain evaluations was higher for the INT-Group vs. SC-Group in the RCT. This is an expression of the standardisation of nursing documentation, which may improve the quality of patient care [12]. The probability of receiving extra opioids for postoperative pain relief is significantly higher if a pain score is documented [8]. Absence of pain assessment, absence of documentation and lack of protocols were among the problems identified in a European survey report from 2008, including 746 hospitals which concluded with postoperative pain management being suboptimal [22].

The majority of nurses agreed that using the ESS and WPM improved collaboration with the physicians. This finding is consistent with one of the goals for developing the ESS, namely to improve communication, thus leading to more accessible help and guidance for the nursing staff [15]. Objective measurements can make the nurses more confident in their clinical judgment, decision-making, and reporting. Also, communication between nurses and doctors may be more to-the-point when using objective signs of patient safety and well-being. The results from our RCT support this; the intervention patients got significantly more on-demand opioids and better pain control on an individual basis than standard care patients [16]. The call-out algorithm of the ESS (ESS ≥ 10) was also used twice in the RCT when the nurses identified patients with severe postoperative pain needing establishing/re-establishing nerve blocks [16].

A vast majority of the nurses agreed that the patients perceive monitoring with ESS and WPM as a positive measure. This finding is consistent with the RCT: all patients in the intervention group reported being satisfied/very satisfied with continuous monitoring at the ward similar to the PACU, significantly better than those receiving standard care [16].

When most of the nurses agreed with the subjective statement that monitoring with ESS and WPM takes extra time compared to standard practice, this is in conflict with the results of the objective recording in the simulation setting. The time consumption in our simulation setting of 4.5 min performing manually standard practice is comparable to previous reports [21, 23]. This misconception of more time consumption may be due to requiring more attention to the new equipment and electronic documentation on top of daily activities. Also, in the simulation setting, the nurses had experience from several previous sessions with patients with ESS and WPM intervention. Further, the setting was different, with no confounding clinical disturbances, and the monitoring equipment was already attached to the patient and connected to the PSE. Interestingly, despite the perception of extra time spent with the intervention, 75% said this intervention improved their overall working situation, and 96% wanted the intervention to continue.

The three cases commented in the questionnaire with delayed diagnosis of non-serious patient issues due to technical problems with the equipment underline the need for further refinement of technology and even better personnel training before these systems become routine. In addition, redundancy in the wearable technical solutions with multiple sensor parameters and automated trend and pattern recognition are areas of future development, identifying deteriorated patients even before any single value reaches the alarm threshold [13].

### Limitations

A limitation of this study is that we could not use a complete, validated questionnaire for the survey. A search for such an instrument was unsuccessful. We ended up selecting items from three validated questionnaires, adapting the questions to our setting, and combining them into one questionnaire. The authors controlled and edited this development process, which induced risk for subjective bias. The need to use a specific and to-the-point questionnaire for this evaluation by busy nurses, not risking a high non-response rate, was the reason for not choosing a generic questionnaire. Ideally, we should have performed structured interviews before developing the questionnaire to establish face validity and reduce risk of subjective bias. Another limitation of this study is the lack of a pilot test to measure internal consistency, regarded as essential for survey instruments [24]. This was not performed in our setting due to the customised and specific questions of the ESS and WPM systems.

Only 28 nurses have experience with this intervention. The low number of eligible participants is a limitation for this study, and the potential of behavioural contagion for people working closely with joint tasks is imminent. A delay of five weeks from the RCT ended until the survey started may have caused a bias towards inaccurate recall of details from the study period. Logistic challenges for the study group caused this delay. The RCT study lasted for seven months, and the nurses took part in multiple patient sessions using the new intervention, which may argue that the delay might not be decisive. However, the delay did not affect the possibility to ask all the involved nurses to participate. The high response rate of 86% reflects this and is a strength of this study. Non-response bias is low but not negligible, with four out of 30 eligible nurses not being given a response. Another strength is that the study design made it possible to collect answers in nurses before being biased by the RCT results.

The small number of participants may have led to a higher variation of responses. However, due to the study design, with only one ward involved, the number of nurses employed at the ward during the randomised trial period was limited. Still, this may limit the generalisability of our findings.

## Conclusions

The combination of the ESS and the WPM systems was perceived as positive by participating nurses and should be developed further for routine clinical care at post-surgical wards. In a simulated clinical test situation to measure time consumption and workload, the ESS and pre-attached WPM systems require less time than the conventional standard of care, and may allow for more frequent clinical monitoring at the post-surgical ward.

### Supplementary information

Below is the link to the electronic supplementary material.

Supplementary material 1 (DOCX 16.2 kb)
