# Supplementary material for: Wireless patient monitoring and Efficacy Safety Score in postoperative treatment at the ward: evaluation of time consumption and usability
Source: J Clin Monit Comput. 2023 Jul 17;38(1):157–64. doi: 10.1007/s10877-023-01053-x (PMC10879331; doi:10.1007/s10877-023-01053-x)
Supplement: Supplementary file 1 — Supplementary material 1 (DOCX 16.2 kb) [file 10877_2023_1053_MOESM1_ESM.docx]

Attachment - Questionnaire survey

1. How will you characterize your knowledge of post-operative pain management?

Very poor – Poor - Neutral - Good - Very good

1. I give my patients good and adequate post-operative pain management

Strongly disagree - Somewhat disagree – Neutral - Somewhat agree - Strongly agree

1. I feel secure in postoperative pain management situations

Strongly disagree - Somewhat disagree – Neutral - Somewhat agree - Strongly agree

1. My documentation of postoperative pain management is good

Strongly disagree - Somewhat disagree – Neutral - Somewhat agree - Strongly agree

1. The patients at my ward receive good and adequate postoperative pain management

Strongly disagree - Somewhat disagree – Neutral - Somewhat agree - Strongly agree

1. Have you treated patients who have been on wireless monitoring with WPM-tablets and Efficacy Safety Score?

Yes/No

1. Monitoring with WPM and ESS provides increased safety for the patients

Strongly disagree - Somewhat disagree – Neutral - Somewhat agree - Strongly agree

1. Monitoring with WPM and ESS provide better post-operative quality for patients compared to standard practice

Strongly disagree - Somewhat disagree – Neutral - Somewhat agree - Strongly agree

1. Monitoring with WPM and ESS improves collaboration with physicians on measures and regulations for medicines

Strongly disagree - Somewhat disagree – Neutral - Somewhat agree - Strongly agree

1. Monitoring with WPM and ESS is perceived as a good measure by the patients

Strongly disagree - Somewhat disagree – Neutral - Somewhat agree - Strongly agree

1. Monitoring with WPM and ESS provides improved confidence for me as a nurse

Strongly disagree - Somewhat disagree – Neutral - Somewhat agree - Strongly agree

1. Monitoring with WPM and ESS takes extra time compared to normal practice

Strongly disagree - Somewhat disagree – Neutral - Somewhat agree - Strongly agree

1. Monitoring with WPM and ESS improves my overall working situation.

Strongly disagree - Somewhat disagree – Neutral - Somewhat agree - Strongly agree

1. How satisfied or dissatisfied are you with the wireless equipment connected to WPM-tablet?

Very dissatisfied – Dissatisfied – Neutral – Satisfied - Very satisfied

1. How satisfied or dissatisfied are you with the WPM-tablet screen layout?

Very dissatisfied – Dissatisfied – Neutral – Satisfied - Very satisfied

1. How satisfied or dissatisfied are you with the WPM training?

Very dissatisfied – Dissatisfied – Neutral – Satisfied - Very satisfied

1. How satisfied or dissatisfied are you with ESS training?

Very dissatisfied – Dissatisfied – Neutral – Satisfied - Very satisfied

1. Have you experienced that WPM and ESS have not detected medical deteriorated patients? Numbers?

Yes/No

1. Have you had medical deteriorated patients you think should have been monitored with tablets and ESS? Numbers?

Yes/No

1. Do you want to continue with WPM and ESS at the ward?

Yes/No

1. What is the best with the project with WPM and ESS?
2. Any other comments to the project?
3. How long work experience do you have?
4. Age?
5. Female/Male
